# Crystal structure of 4-bromo-3-[(5-bromo­thio­phen-2-yl)methyl­idene]-2-(di­cyano­methyl­idene)-5,6-di­fluoro-2,3-di­hydro­inden-1-one

**DOI:** 10.1107/S2056989026004469

**Published:** 2026-05-07

**Authors:** Sunyu Cao, Xiaofeng Chen, Zhipeng Yu

**Affiliations:** a Institutes of Physical Science and Information Technology Anhui University Hefei People’s Republic of China; b Zhejiang University,Hangzhou People’s Republic of China; Vienna University of Technology, Austria

**Keywords:** crystal structure, di­cyano­methyl­ene indenone, bromo­thio­phene, pi-conjugated system, halogen bond (Br⋯O), C—H⋯N hydrogen bond

## Abstract

In the mol­ecular structure of the title compound, C_17_H_4_Br_2_F_2_N_2_OS, the indenone moiety is close to planar [the dihedral angle between the phenyl and the five-membered ring plane is 4.2 (4)°], and the thio­phene ring is only slightly inclined to this fragment. In the extended structure, a short Br⋯O contact considered as a halogen bond and a weak C—H⋯N inter­action contribute to the crystal packing.

## Abstract 2

In the crystal, the nearly planar title mol­ecules are held together by a Br⋯O halogen bond and a C—H⋯N inter­action.

## 1. Chemical context

Polyhalogenation is a convenient strategy for tuning the properties of π-conjugated organic building blocks, because halogen substituents can be introduced without altering the underlying conjugated framework while still allowing systematic modulation of the electronic structure and crystal packing (Baker *et al.*, 2012 ▸; Facchetti, 2011 ▸). In particular, combinations of heavier and lighter halogens (*e.g.* Br and F) can influence the mol­ecular electrostatic potential and polarizability, and may facilitate directional inter­molecular contacts, including halogen bonding, which contribute to the definition of packing motifs (Metrangolo & Resnati, 2001 ▸; Cavallo *et al.*, 2016 ▸; Desiraju *et al.*, 2013 ▸). Such effects are especially relevant for donor⋯acceptor-type conjugated mol­ecules, in which optical and charge-transport properties can be sensitive to subtle changes in the inter­molecular arrangement (Coropceanu *et al.*, 2007 ▸; Sirringhaus, 2014 ▸).
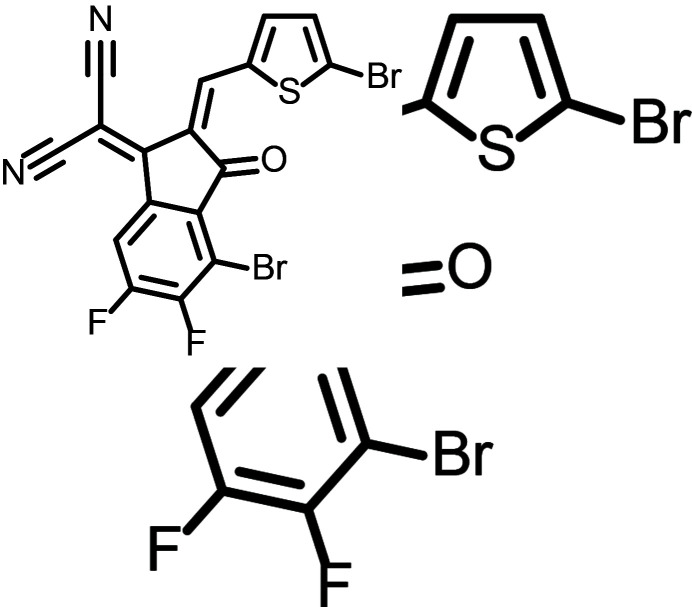


The title compound, C_17_H_4_Br_2_F_2_N_2_OS, comprises an electron-withdrawing di­cyano­methyl­ene fragment and a carbonyl group within a conjugated indanone-based framework, tog­ether with a multi-halogenated substitution pattern that is frequently employed in the design of electron-deficient chromophores (Lin & Zhan, 2016 ▸). Although no device performance data are reported here, determination of the crystal structure is useful for assessing the conformational preferences of the conjugated skeleton and for identifying the inter­molecular contacts promoted by the Br/F substitution.

## 2. Structural commentary

**Figure 1:**
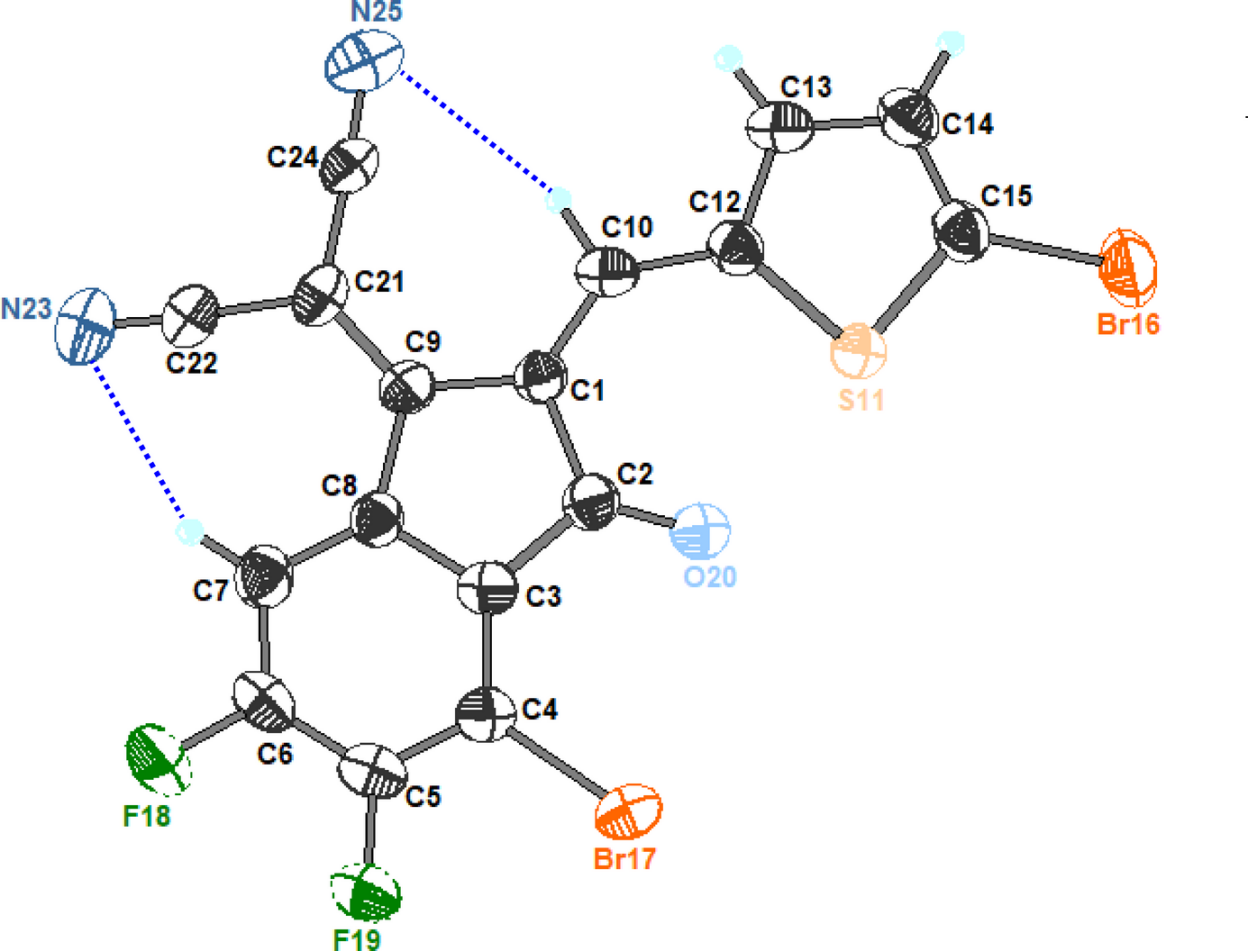
The mol­ecular structure of the title compound with displacement ellipsoids drawn at the 50% probability level. H atoms are shown as small spheres of arbitrary radius. C—H⋯N hydrogen bonds are shown as blue dashed lines.

The mol­ecular structure of the title compound is shown in Fig. 1 ▸. The mol­ecule consists of a di­cyano­methyl­ene-substituted 2,3-di­hydro-1*H*-inden-1-one (indenone) unit that is connected to a 5-bromo­thio­phene ring through an exocyclic C=C linkage involving atoms C1, C10 and C12. In the resulting π-conjugated mol­ecule, the indenone carbonyl group and the di­cyano­methyl­ene fragment form an electron-deficient core, while the thienyl substituent further extends the conjugation. In line with the materials-guided use of multi-halogenation, the presence of two bromine and two fluorine atoms may provide electronic tuning as well as potential sites for structure-directing inter­molecular contacts in the solid state.

The indenone ring system is close to planar. The phenyl ring (C3–C8; r.m.s. deviation = 0.011 Å) and the five-membered ring (C1/C2/C3/C8/C9; r.m.s. deviation = 0.008 Å) form a dihedral angle of 4.2 (4)°. The thio­phene ring P (S11/C12–C15; r.m.s. deviation = 0.005 Å) is slightly twisted with respect to the indenone core, making dihedral angles of 7.5 (3)° with the phenyl ring and 5.4 (3)° with the five-membered ring. The near-coplanar arrangement across the linking fragments is supported by the torsion angles C9—C1—C10—C12 [–176.6 (7)°] and C1—C10—C12—S1 [3.7 (13)°]. In the di­cyano­methyl­ene substituent, torsion angles C1—C9—C21—C22 [173.2 (7)°] and C1—C9—C21—C24 [–6.3 (11)°] indicate an overall nearly planar conjugated skeleton with a small asymmetry in the orientations of the two cyano groups. The mol­ecular conformation is stabilized by two weak C—H⋯N intra­molecular hydrogen bonds (entries 1 and 2 in Table 1 ▸).

## 3. Supra­molecular features

**Figure 2:**
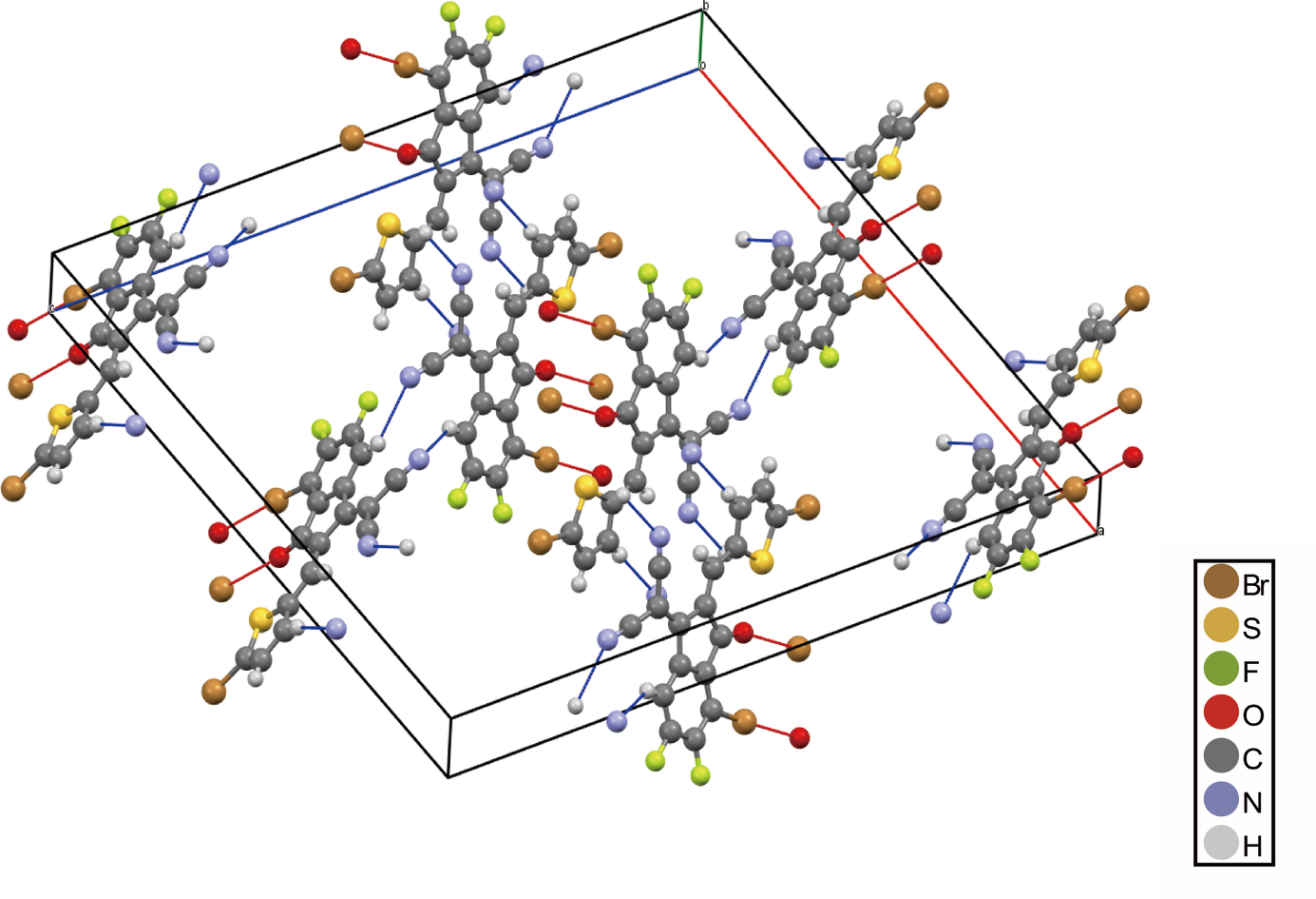
Crystal packing of the title compound viewed approximately along the *b* axis. Inter­molecular Br⋯O contacts are shown as red lines, and inter­molecular C—H⋯N inter­actions as blue lines.

In the extended structure, a short and highly directional inter­molecular Br⋯O contact involving the carbonyl O atom is present. As shown in Fig. 2 ▸, this halogen bond (Cavallo *et al.*, 2016 ▸; Desiraju *et al.*, 2013 ▸) is nearly linear [Br17⋯O20^i^ = 3.141 (5) Å; C4—Br17⋯O20^i^ = 177.6 (2)°; symmetry code: (i) 1 − *x*, −*y*, 1 − *z*] and connects adjacent mol­ecules into a centrosymmetric dimer.

In addition, a weak C—H⋯N inter­action involving the phenyl ring and one of the cyano­methyl­ene N atoms is present (entry 3 in Table 1 ▸) that may help to consolidate the crystal packing.

## 4. Database survey

A substructure search of the Cambridge Structural Database (CSD: version 2026.1; Groom *et al.*, 2016 ▸) was carried out for neutral mol­ecules containing the same conjugated indenone/di­cyano­methyl­ene framework as the title compound. The search returned 51 hits. Representative closely related structures include CAPYUN (Popova *et al.*, 1983 ▸), IDOYUW (Palusiak *et al.*, 2006 ▸), RAZMEM and RAZLUB (Capobianco *et al.*, 2012 ▸), TETVAT (Shao *et al.*, 2023 ▸), PAWMUZ (Terenti *et al.*, 2022 ▸), SOFPOT and SOFPUZ (Masuda *et al.*, 2008 ▸) and XAKJAX (Francos *et al.*, 2016 ▸).

Structural variations are mainly associated with the substituents on the indenone ring and the exocyclic ar­yl(heteroar­yl)methyl­idene fragment, including differences in halogen substitution. In comparison with these related compounds, the title mol­ecule bears two Br atoms (on the indenone ring and the thio­phene ring) together with two F atoms on the fused benzene ring. Such substitutions change the steric demand and the distribution of electron density around the carbonyl and nitrile groups, which can influence the balance of weak inter­molecular contacts. Consistent with this, the crystal structure of the title compound is primarily stabilized by C—H⋯N inter­actions involving the nitrile N atoms and by a directional Br⋯O contact involving the carbonyl O atom; the latter inter­action is enabled by the presence and orientation of the bromine substituent and is not necessarily present in all related structures lacking an appropriately positioned halogen donor.

## 5. Synthesis and crystallization

**Figure 3:**
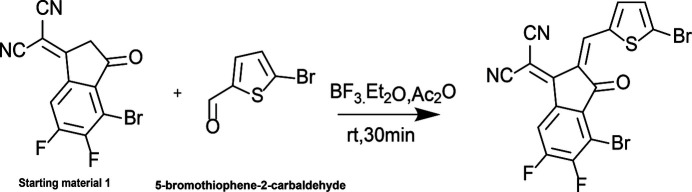
Synthesis scheme of the title compound.

The synthesis scheme to obtain the title compound is given in Fig. 3 ▸. Starting material 1 (40 mg, 0.129 mmol) and 5-bromo­thio­phene-2-carbaldehyde (31 mg, 0.162 mmol) were dissolved in 1,2-di­chloro­ethane (12 ml). Tri­fluoro­boric acid diethyl etherate (BF_3_·Et_2_O, 0.10 ml) and acetic anhydride (Ac_2_O, 0.10 ml) were added, and the reaction mixture was stirred at room temperature for 30 min. The mixture was then extracted with di­chloro­methane and the combined organic layers dried over anhydrous Na_2_SO_4_ and concentrated under reduced pressure. The crude product was purified by column chromatography using chloro­form as eluent to afford the title compound as an orange–red solid (44 mg, 0.091 mmol, 71% based on starting material **1**. The product was characterized by ^1^H NMR spectroscopy (details given in the electronic supplementary information). Single crystals suitable for X-ray diffraction were obtained by gas-liquid diffusion of *n*-hexane into a di­chloro­methane solution of the product over 2 d at room temperature.

## 6. Refinement

Crystal data, data collection and structure refinement details are summarized in Table 2 ▸. Hydrogen atoms were placed in calculated positions and refined using a riding model [C—H = 0.93 Å, *U*_iso_(H) = 1.2*U*_eq_(C)]. Six reflections, −20 4 18, 14 2 12, −5 3 21, −8 2 25, −9 1 28 and 12 4 11, were omitted as clear outliers. The maximum and minimum residual electron-density peaks are 1.70 and 0.97 Å, respectively, from atom Br17.

## Supplementary Material

Crystal structure: contains datablock(s) I. DOI: 10.1107/S2056989026004469/wm5797sup1.cif

Structure factors: contains datablock(s) I. DOI: 10.1107/S2056989026004469/wm5797Isup2.hkl

1H NMR spectra. DOI: 10.1107/S2056989026004469/wm5797sup4.tif

Supporting information file. DOI: 10.1107/S2056989026004469/wm5797Isup4.cml

CCDC reference: 2543339

Additional supporting information:  crystallographic information; 3D view; checkCIF report

## Figures and Tables

**Table 1:** Hydrogen-bond geometry (Å, °)

*D*—H⋯*A*	*D*—H	H⋯*A*	*D*⋯*A*	*D*—H⋯*A*
C7—H7⋯N23	0.93	2.59	3.394 (12)	145
C10—H10⋯N25	0.93	2.61	3.489 (10)	159
C13—H13⋯N25^i^	0.93	2.48	3.405 (11)	171

Symmetry code: (i) 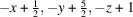.

**Table 2:** Experimental details

Crystal data	
Chemical formula	C_17_H_4_Br_2_F_2_N_2_OS
*M*_r_	482.08
Crystal system, space group	Monoclinic, *C*2/*c*
Temperature (K)	293
*a*, *b*, *c* (Å)	22.9059 (8), 5.6693 (2), 25.9096 (9)
β (°)	108.971 (4)
*V* (Å^3^)	3181.9 (2)
*Z*	8
Radiation type	Cu *K*α
μ (mm^−1^)	7.98
Crystal size (mm)	0.25 × 0.20 × 0.20

Data collection	
Diffractometer	XtaLAB Synergy R, HyPix
Absorption correction	Multi-scan (*CrysAlis PRO*; Rigaku OD, 2024 ▸)
*T*_min_, *T*_max_	0.116, 1.000
No. of measured, independent and observed [*I* > 2σ(*I*)] reflections	9183, 3095, 2432
*R*_int_	0.047
(sin θ/λ)_max_ (Å^−1^)	0.636

Refinement	
*R*[*F*^2^ > 2σ(*F*^2^)], *wR*(*F*^2^), *S*	0.042, 0.132, 1.15
No. of reflections	3095
No. of parameters	226
H-atom treatment	H-atom parameters constrained
Δρ_max_, Δρ_min_ (e Å^−3^)	0.77, −1.15

Computer programs: *CrysAlis PRO* (Rigaku OD, 2024 ▸), *OLEX2.solve* (Bourhis *et al.*, 2015 ▸), *SHELXL* (Sheldrick, 2015 ▸), *OLEX2* (Dolomanov *et al.*, 2009 ▸) and *publCIF* (Westrip, 2010 ▸).

## supplementary crystallographic information

### 2-{4-Bromo-3-[(E)-(5-bromothiophen-2-yl)methylidene]-5,6-difluoro-1-oxoinden-2-ylidene}propanedinitrile Crystal data

**Table:**

C_17_H_4_Br_2_F_2_N_2_OS	*F*(000) = 1856
*M**_r_* = 482.08	*D*_x_ = 2.013 Mg m^−^^3^
Monoclinic, *C*2/*c*	Cu *K*α radiation, λ = 1.54184 Å
*a* = 22.9059 (8) Å	Cell parameters from 4587 reflections
*b* = 5.6693 (2) Å	θ = 3.6–76.1°
*c* = 25.9096 (9) Å	µ = 7.98 mm^−^^1^
β = 108.971 (4)°	*T* = 293 K
*V* = 3181.9 (2) Å^3^	Block, orange-red
*Z* = 8	0.25 × 0.20 × 0.20 mm

### 2-{4-Bromo-3-[(E)-(5-bromothiophen-2-yl)methylidene]-5,6-difluoro-1-oxoinden-2-ylidene}propanedinitrile Data collection

**Table:**

XtaLAB Synergy R, HyPix diffractometer	2432 reflections with *I* > 2σ(*I*)
Detector resolution: 10.0000 pixels mm^-1^	*R*_int_ = 0.047
ω scans	θ_max_ = 78.8°, θ_min_ = 3.6°
Absorption correction: multi-scan (CrysAlisPro; Rigaku OD, 2024)	*h* = −27→28
*T*_min_ = 0.116, *T*_max_ = 1.000	*k* = −5→7
9183 measured reflections	*l* = −32→32
3095 independent reflections	

### 2-{4-Bromo-3-[(E)-(5-bromothiophen-2-yl)methylidene]-5,6-difluoro-1-oxoinden-2-ylidene}propanedinitrile Refinement

**Table:**

Refinement on *F*^2^	Primary atom site location: iterative
Least-squares matrix: full	Hydrogen site location: inferred from neighbouring sites
*R*[*F*^2^ > 2σ(*F*^2^)] = 0.042	H-atom parameters constrained
*wR*(*F*^2^) = 0.132	*w* = 1/[σ^2^(*F*_o_^2^) + (0.015*P*)^2^ + 49.4493*P*] where *P* = (*F*_o_^2^ + 2*F*_c_^2^)/3
*S* = 1.15	(Δ/σ)_max_ < 0.001
3095 reflections	Δρ_max_ = 0.77 e Å^−^^3^
226 parameters	Δρ_min_ = −1.15 e Å^−^^3^
0 restraints	

### 2-{4-Bromo-3-[(E)-(5-bromothiophen-2-yl)methylidene]-5,6-difluoro-1-oxoinden-2-ylidene}propanedinitrile Special details

**Table:**

Geometry. All esds (except the esd in the dihedral angle between two l.s. planes) are estimated using the full covariance matrix. The cell esds are taken into account individually in the estimation of esds in distances, angles and torsion angles; correlations between esds in cell parameters are only used when they are defined by crystal symmetry. An approximate (isotropic) treatment of cell esds is used for estimating esds involving l.s. planes.

### 2-{4-Bromo-3-[(E)-(5-bromothiophen-2-yl)methylidene]-5,6-difluoro-1-oxoinden-2-ylidene}propanedinitrile Fractional atomic coordinates and isotropic or equivalent isotropic displacement parameters (Å^2^)

**Table:**

	*x*	*y*	*z*	*U*_iso_*/*U*_eq_	
Br17	0.54902 (3)	0.06639 (13)	0.57194 (3)	0.0452 (2)	
Br16	0.25776 (5)	0.26446 (16)	0.29651 (3)	0.0663 (3)	
S11	0.33207 (7)	0.4103 (3)	0.41385 (6)	0.0407 (4)	
F18	0.62344 (18)	0.2168 (8)	0.68610 (17)	0.0589 (11)	
F19	0.6063 (2)	0.5718 (9)	0.74651 (17)	0.0664 (13)	
O20	0.4295 (2)	0.3420 (9)	0.5018 (2)	0.0554 (13)	
C1	0.3949 (3)	0.6807 (11)	0.5405 (3)	0.0357 (13)	
C3	0.4832 (3)	0.4643 (11)	0.5943 (3)	0.0370 (14)	
C4	0.5329 (3)	0.3126 (11)	0.6139 (3)	0.0376 (14)	
N25	0.3078 (3)	1.2489 (12)	0.5599 (3)	0.0602 (17)	
C2	0.4346 (3)	0.4760 (11)	0.5400 (3)	0.0384 (14)	
C9	0.4182 (3)	0.7844 (11)	0.5951 (3)	0.0358 (13)	
C21	0.3949 (3)	0.9738 (11)	0.6149 (3)	0.0402 (15)	
C12	0.3154 (3)	0.6618 (11)	0.4446 (3)	0.0362 (13)	
C8	0.4730 (3)	0.6469 (11)	0.6271 (2)	0.0357 (13)	
C10	0.3448 (3)	0.7501 (11)	0.4982 (3)	0.0376 (14)	
H10	0.325749	0.883990	0.505978	0.045*	
C24	0.3456 (3)	1.1198 (11)	0.5828 (3)	0.0399 (14)	
C13	0.2659 (3)	0.7793 (12)	0.4081 (3)	0.0451 (16)	
H13	0.249940	0.918681	0.416920	0.054*	
C7	0.5150 (3)	0.6862 (13)	0.6790 (3)	0.0459 (16)	
H7	0.509451	0.809712	0.700609	0.055*	
C5	0.5740 (3)	0.3538 (13)	0.6655 (3)	0.0443 (16)	
C15	0.2725 (3)	0.4711 (12)	0.3549 (3)	0.0429 (15)	
C14	0.2420 (3)	0.6726 (13)	0.3572 (3)	0.0464 (16)	
H14	0.209246	0.732103	0.328524	0.056*	
C6	0.5642 (3)	0.5386 (13)	0.6972 (3)	0.0460 (16)	
N23	0.4306 (4)	1.1109 (15)	0.7145 (3)	0.082 (2)	
C22	0.4170 (3)	1.0470 (13)	0.6707 (3)	0.0498 (17)	

### 2-{4-Bromo-3-[(E)-(5-bromothiophen-2-yl)methylidene]-5,6-difluoro-1-oxoinden-2-ylidene}propanedinitrile Atomic displacement parameters (Å^2^)

**Table:**

	*U*^11^	*U*^22^	*U*^33^	*U*^12^	*U*^13^	*U*^23^
Br17	0.0427 (4)	0.0407 (4)	0.0553 (4)	0.0068 (3)	0.0203 (3)	0.0006 (3)
Br16	0.0885 (7)	0.0584 (5)	0.0404 (4)	−0.0060 (5)	0.0049 (4)	−0.0067 (4)
S11	0.0414 (8)	0.0371 (8)	0.0388 (8)	0.0041 (7)	0.0064 (7)	−0.0021 (7)
F18	0.047 (2)	0.070 (3)	0.051 (2)	0.015 (2)	0.0043 (19)	0.004 (2)
F19	0.063 (3)	0.075 (3)	0.045 (2)	0.004 (2)	−0.006 (2)	−0.001 (2)
O20	0.054 (3)	0.054 (3)	0.048 (3)	0.017 (2)	0.001 (2)	−0.011 (2)
C1	0.035 (3)	0.032 (3)	0.039 (3)	0.002 (3)	0.011 (3)	0.000 (3)
C3	0.032 (3)	0.033 (3)	0.045 (3)	−0.002 (3)	0.011 (3)	0.003 (3)
C4	0.036 (3)	0.038 (3)	0.041 (3)	0.002 (3)	0.016 (3)	0.005 (3)
N25	0.064 (4)	0.047 (4)	0.076 (5)	0.011 (3)	0.031 (4)	0.003 (4)
C2	0.033 (3)	0.035 (3)	0.044 (4)	−0.003 (3)	0.008 (3)	−0.005 (3)
C9	0.038 (3)	0.030 (3)	0.040 (3)	−0.003 (3)	0.014 (3)	0.000 (3)
C21	0.046 (4)	0.033 (3)	0.045 (4)	−0.004 (3)	0.020 (3)	−0.007 (3)
C12	0.032 (3)	0.037 (3)	0.037 (3)	−0.003 (3)	0.007 (2)	0.000 (3)
C8	0.038 (3)	0.036 (3)	0.034 (3)	0.000 (3)	0.014 (3)	0.000 (3)
C10	0.034 (3)	0.032 (3)	0.046 (4)	0.003 (3)	0.011 (3)	0.006 (3)
C24	0.047 (4)	0.031 (3)	0.046 (4)	0.002 (3)	0.022 (3)	−0.002 (3)
C13	0.042 (4)	0.038 (4)	0.053 (4)	0.009 (3)	0.012 (3)	0.008 (3)
C7	0.054 (4)	0.045 (4)	0.041 (4)	0.000 (3)	0.019 (3)	0.001 (3)
C5	0.036 (3)	0.046 (4)	0.047 (4)	0.002 (3)	0.009 (3)	0.010 (3)
C15	0.045 (4)	0.040 (4)	0.038 (3)	−0.007 (3)	0.006 (3)	−0.001 (3)
C14	0.049 (4)	0.047 (4)	0.039 (3)	0.002 (3)	0.008 (3)	0.007 (3)
C6	0.040 (3)	0.052 (4)	0.038 (3)	−0.004 (3)	0.002 (3)	0.006 (3)
N23	0.096 (6)	0.090 (6)	0.057 (4)	0.026 (5)	0.018 (4)	−0.022 (4)
C22	0.058 (4)	0.045 (4)	0.047 (4)	0.008 (4)	0.017 (3)	−0.008 (3)

### 2-{4-Bromo-3-[(E)-(5-bromothiophen-2-yl)methylidene]-5,6-difluoro-1-oxoinden-2-ylidene}propanedinitrile Geometric parameters (Å, º)

**Table:**

Br17—C4	1.877 (7)	C9—C8	1.480 (9)
Br16—C15	1.855 (7)	C21—C24	1.428 (9)
S11—C12	1.735 (7)	C21—C22	1.428 (9)
S11—C15	1.720 (7)	C12—C10	1.422 (9)
F18—C5	1.333 (8)	C12—C13	1.389 (9)
F19—C6	1.341 (7)	C8—C7	1.395 (9)
O20—C2	1.223 (8)	C10—H10	0.9300
C1—C2	1.477 (9)	C13—H13	0.9300
C1—C9	1.464 (8)	C13—C14	1.391 (9)
C1—C10	1.362 (8)	C7—H7	0.9300
C3—C4	1.385 (9)	C7—C6	1.359 (10)
C3—C2	1.485 (9)	C5—C6	1.394 (10)
C3—C8	1.405 (9)	C15—C14	1.352 (10)
C4—C5	1.382 (9)	C14—H14	0.9300
N25—C24	1.142 (9)	N23—C22	1.136 (9)
C9—C21	1.372 (9)		
			
C15—S11—C12	90.8 (3)	C7—C8—C9	130.5 (6)
C9—C1—C2	106.9 (5)	C1—C10—C12	133.8 (6)
C10—C1—C2	125.5 (6)	C1—C10—H10	113.1
C10—C1—C9	127.6 (6)	C12—C10—H10	113.1
C4—C3—C2	130.4 (6)	N25—C24—C21	175.0 (8)
C4—C3—C8	121.1 (6)	C12—C13—H13	122.6
C8—C3—C2	108.5 (5)	C12—C13—C14	114.9 (6)
C3—C4—Br17	122.9 (5)	C14—C13—H13	122.6
C5—C4—Br17	119.1 (5)	C8—C7—H7	121.0
C5—C4—C3	117.9 (6)	C6—C7—C8	118.0 (7)
O20—C2—C1	126.7 (6)	C6—C7—H7	121.0
O20—C2—C3	125.7 (6)	F18—C5—C4	120.7 (6)
C1—C2—C3	107.5 (5)	F18—C5—C6	118.7 (6)
C1—C9—C8	107.8 (5)	C4—C5—C6	120.5 (6)
C21—C9—C1	127.7 (6)	S11—C15—Br16	118.7 (4)
C21—C9—C8	124.4 (6)	C14—C15—Br16	127.6 (5)
C9—C21—C24	124.4 (6)	C14—C15—S11	113.7 (5)
C9—C21—C22	123.5 (6)	C13—C14—H14	124.5
C22—C21—C24	112.0 (6)	C15—C14—C13	111.1 (6)
C10—C12—S11	129.2 (5)	C15—C14—H14	124.5
C13—C12—S11	109.5 (5)	F19—C6—C7	120.2 (7)
C13—C12—C10	121.3 (6)	F19—C6—C5	117.6 (6)
C3—C8—C9	109.1 (5)	C7—C6—C5	122.2 (6)
C7—C8—C3	120.1 (6)	N23—C22—C21	175.2 (8)
			
Br17—C4—C5—F18	−2.0 (9)	C2—C3—C8—C7	−175.3 (6)
Br17—C4—C5—C6	178.5 (5)	C9—C1—C2—O20	179.2 (7)
Br16—C15—C14—C13	179.4 (5)	C9—C1—C2—C3	−2.0 (7)
S11—C12—C10—C1	3.7 (11)	C9—C1—C10—C12	−176.6 (7)
S11—C12—C13—C14	0.0 (8)	C9—C8—C7—C6	−175.4 (6)
S11—C15—C14—C13	−1.4 (8)	C21—C9—C8—C3	179.2 (6)
F18—C5—C6—F19	1.7 (10)	C21—C9—C8—C7	−7.0 (11)
F18—C5—C6—C7	179.8 (6)	C12—S11—C15—Br16	−179.5 (4)
C1—C9—C21—C24	−6.3 (11)	C12—S11—C15—C14	1.2 (6)
C1—C9—C21—C22	173.2 (7)	C12—C13—C14—C15	0.9 (9)
C1—C9—C8—C3	−0.6 (7)	C8—C3—C4—Br17	−179.8 (5)
C1—C9—C8—C7	173.2 (7)	C8—C3—C4—C5	−3.3 (9)
C3—C4—C5—F18	−178.6 (6)	C8—C3—C2—O20	−179.5 (7)
C3—C4—C5—C6	1.9 (10)	C8—C3—C2—C1	1.7 (7)
C3—C8—C7—C6	−2.2 (10)	C8—C9—C21—C24	174.0 (6)
C4—C3—C2—O20	1.9 (12)	C8—C9—C21—C22	−6.5 (10)
C4—C3—C2—C1	−176.9 (6)	C8—C7—C6—F19	178.8 (6)
C4—C3—C8—C9	178.1 (6)	C8—C7—C6—C5	0.8 (11)
C4—C3—C8—C7	3.5 (9)	C10—C1—C2—O20	1.6 (11)
C4—C5—C6—F19	−178.8 (6)	C10—C1—C2—C3	−179.7 (6)
C4—C5—C6—C7	−0.7 (11)	C10—C1—C9—C21	−0.6 (11)
C2—C1—C9—C21	−178.1 (6)	C10—C1—C9—C8	179.2 (6)
C2—C1—C9—C8	1.6 (7)	C10—C12—C13—C14	179.8 (6)
C2—C1—C10—C12	0.5 (12)	C13—C12—C10—C1	−176.0 (7)
C2—C3—C4—Br17	−1.3 (10)	C15—S11—C12—C10	179.6 (6)
C2—C3—C4—C5	175.2 (6)	C15—S11—C12—C13	−0.7 (5)
C2—C3—C8—C9	−0.7 (7)		

### 2-{4-Bromo-3-[(E)-(5-bromothiophen-2-yl)methylidene]-5,6-difluoro-1-oxoinden-2-ylidene}propanedinitrile Hydrogen-bond geometry (Å, º)

**Table:**

*D*—H···*A*	*D*—H	H···*A*	*D*···*A*	*D*—H···*A*
C7—H7···N23	0.93	2.59	3.394 (12)	145
C10—H10···N25	0.93	2.61	3.489 (10)	159
C13—H13···N25^i^	0.93	2.48	3.405 (11)	171

Symmetry code: (i) −*x*+1/2, −*y*+5/2, −*z*+1.
