# Supplementary figures and images for: Crystal structure of 4-bromo-3-[(5-bromo­thio­phen-2-yl)methyl­idene]-2-(di­cyano­methyl­idene)-5,6-di­fluoro-2,3-di­hydro­inden-1-one
Source: Acta Crystallogr E Crystallogr Commun. 2026 May 7;82(Pt 6):596–9. doi: 10.1107/S2056989026004469 (PMC13239018; doi:10.1107/S2056989026004469)

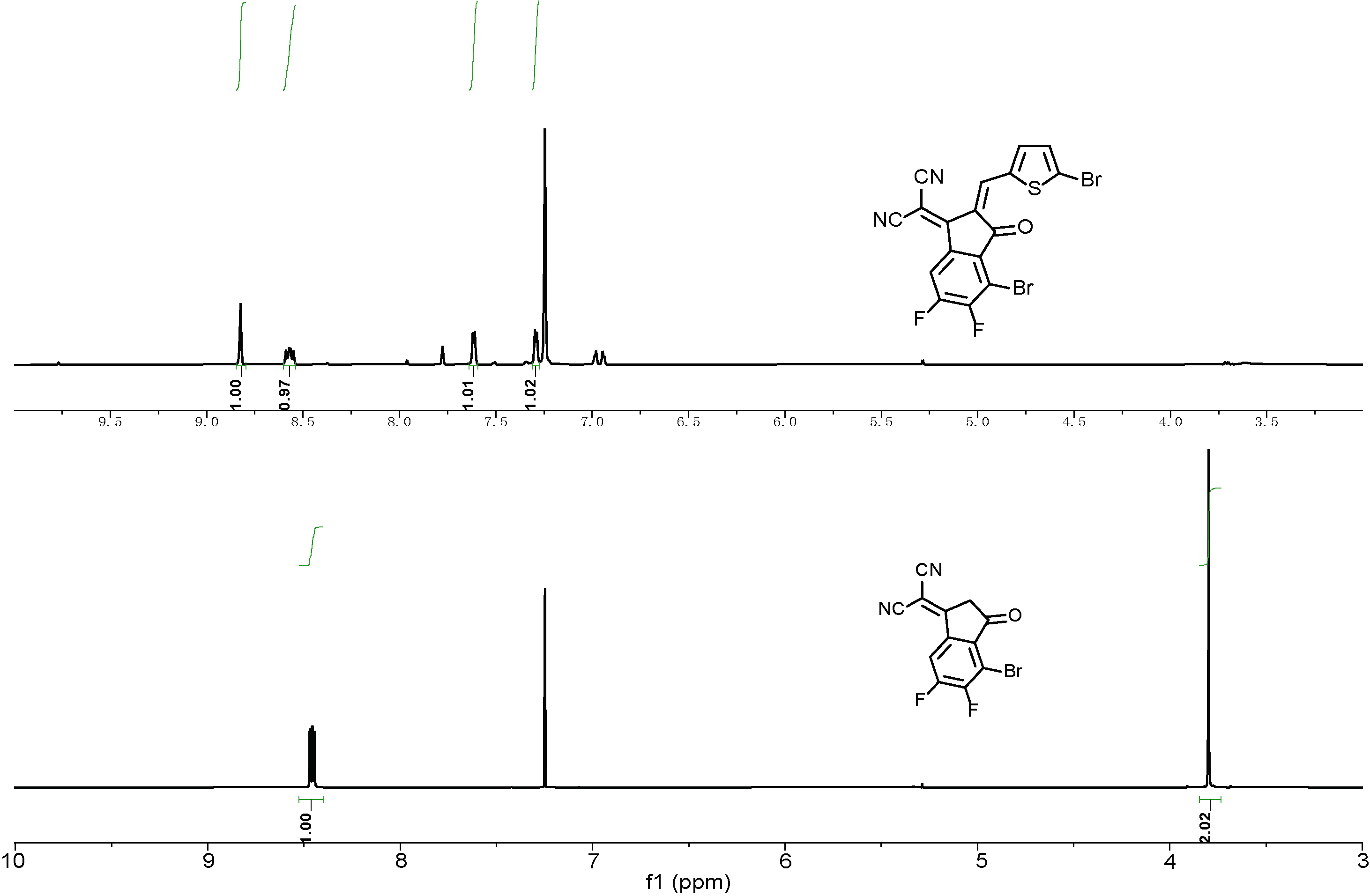

Supplement: Supplementary file 3 [file e-82-00596-sup4.tif]
